# Distinct Changes in cAMP and Extracellular Signal-Regulated Protein Kinase Signalling in L-DOPA-Induced Dyskinesia

**DOI:** 10.1371/journal.pone.0012322

**Published:** 2010-08-23

**Authors:** Emanuela Santini, Veronique Sgambato-Faure, Qin Li, Marc Savasta, Sandra Dovero, Gilberto Fisone, Erwan Bezard

**Affiliations:** 1 Department of Neuroscience, Karolinska Institutet, Stockholm, Sweden; 2 INSERM U836, Grenoble Institut des Neurosciences, Grenoble, France; 3 Centre de Neuroscience Cognitive, Centre National de la Recherche Scientifique UMR 5229 - Université Claude Bernard Lyon I, Bron, France; 4 Université Victor Segalen-Bordeaux 2, Centre National de la Recherche Scientifique, Bordeaux Institute of Neuroscience, UMR 5227, Bordeaux, France; 5 Institute of Lab Animal Sciences, China Academy of Medical Sciences, Beijing, China; National Institutes of Health, United States of America

## Abstract

**Background:**

In rodents, the development of dyskinesia produced by L-DOPA in the dopamine-depleted striatum occurs in response to increased dopamine D1 receptor-mediated activation of the cAMP - protein kinase A and of the Ras-extracellular signal-regulated kinase (ERK) signalling pathways. However, very little is known, in non-human primates, about the regulation of these signalling cascades and their association with the induction, manifestation and/or maintenance of dyskinesia.

**Methodology/Results:**

We here studied, in the gold-standard non-human primate model of Parkinson's disease, the changes in PKA-dependent phosphorylation of DARPP-32 and GluR1 AMPA receptor, as well as in ERK and ribosomal protein S6 (S6) phosphorylation, associated to acute and chronic administration of L-DOPA. Increased phosphorylation of DARPP-32 and GluR1 was observed in both L-DOPA first-ever exposed and chronically-treated dyskinetic parkinsonian monkeys. In contrast, phosphorylation of ERK and S6 was enhanced preferentially after acute L-DOPA administration and decreased during the course of chronic treatment.

**Conclusion:**

Dysregulation of cAMP signalling is maintained during the course of chronic L-DOPA administration, while abnormal ERK signalling peaks during the initial phase of L-DOPA treatment and decreases following prolonged exposure. While cAMP signalling enhancement is associated with dyskinesia, abnormal ERK signalling is associated with priming.

## Introduction

In Parkinson's disease (PD), long-term treatment with L-3,4-dihydroxyphenylalanine (L-DOPA) leads to the emergence of dyskinesia, or involuntary aimless movements [1]. Work in rodents has led to the identification of numerous changes in signalling potentially implicated in L-DOPA-induced dyskinesis (LID) [2]. Prominent among these changes is a marked increase in the dopamine D1 receptor (D1R)-mediated activation of (i) the canonical adenylyl cyclase - protein kinase A (PKA) - dopamine and cAMP-regulated phosphoprotein, 32 kDa (DARPP-32) signalling cascade [3], [4] and (ii) the non-canonical Ras-extracellular signal-regulated kinase (ERK) signalling cascade [5], [6], [7], [8]. In order to identify potential therapeutic targets, it is essential to understand the role of these signalling cascades in the actual induction, manifestation and/or maintenance of LID. However, the correlation between abnormal PKA/DARPP-32 and Ras-ERK signalling and severity of LID has been established only in rodent models of LID and without the appropriate L-DOPA-treated unlesioned controls. This limits a clear-cut association between specific biochemical events and one or several of the above mentioned key steps in LID elicitation.

In this study, we made use of the gold standard macaque model of LIDs [3], [9] to show that dysregulation of PKA/DARPP-32 signalling is mainly associated to the manifestation of dyskinesia, while abnormal Ras-ERK signalling is most evident during the development of this condition.

## Materials and Methods

### Animals

Thirty-nine F2-bred female rhesus monkeys (*Macaca mulatta*, Xierxin, Beijing, PR of China; mean weight 5.3±0.8 kg; mean age  = 5±1 years) were used. Animals were housed in individual primate cages under controlled conditions of humidity (50±5%), temperature (24±1°C) and light (12 h light/12 h dark cycles, time lights on 8:00 am) with food and water *ad libitum*. Experiments were carried out in accordance with European Communities Council Directive of 24 November 1986 (86/609/EEC) for care of laboratory animals in an AAALAC-accredited facility following acceptance of study design by the Institute of Lab Animal Science (Chinese Academy of Science, Beijing, China) IACUC. Veterinarians skilled in the healthcare and maintenance of non-human primates supervised animal care. All reasonable efforts were made to minimise animal suffering. The use of primates was minimised by using an experimental design that permits statistically-significant changes to be demonstrated with the smallest number of animals per group and the smallest number of groups, consistent with scientific rigour. Experiments followed previously published procedures [3], [11], [12], [13], [14]. All animals were killed with a sodium pentobarbital overdose (150 mg/kg, i.v.). Dissection of different brain regions were performed on ice with the brain immersed in cold saline (0.9%) in less than 15 min. The dorsolateral striatum was dissected from each hemisphere, immediately frozen at −45°C in isopentane and then stored at −80°C.

### Experimental Protocol

**Figure 1:**
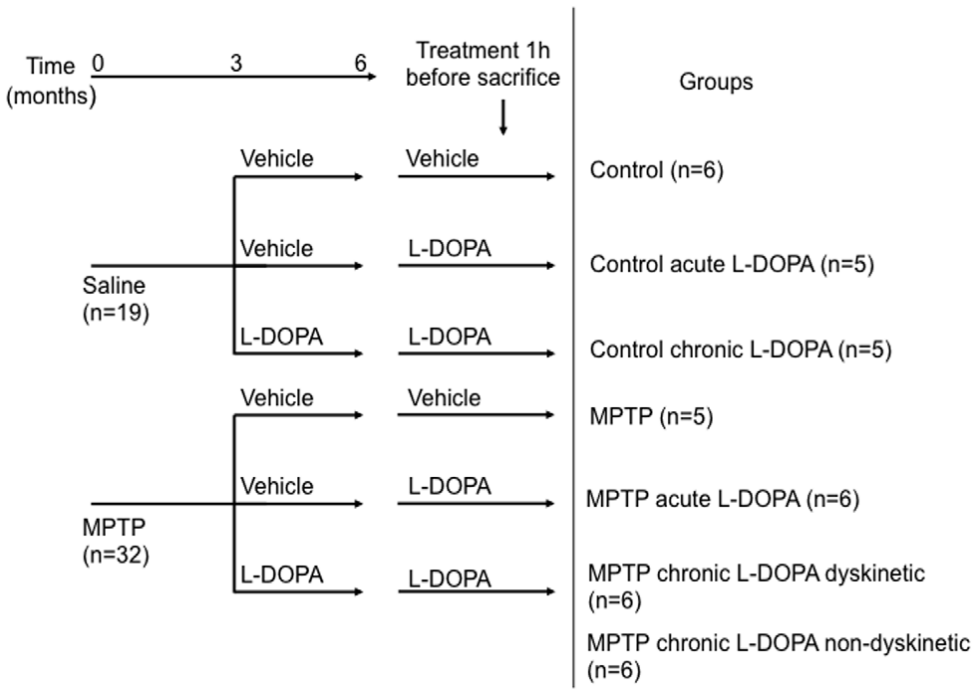
Experimental flowchart illustrating study design, treatments and group assignments.

The experimental flowchart is described in figure 1. Six animals were kept as untreated controls (control group), five received a single dose of 20 mg/kg of L-DOPA p.o (control acute L-DOPA) and five received 20 mg/kg of L-DOPA twice daily for three months (control chronic L-DOPA). The remaining 32 animals were treated daily (9:00 am) with 1-methyl-4-phenyl 1,2,3,6-tetrahydropyridine (MPTP) hydrochloride (0.2 mg/kg, i.v., Sigma, St Louis, MO) dissolved in saline according to a previously described protocol [10]. Following stabilization of the MPTP-induced syndrome, animals received saline (MPTP; n = 5), a single dose of 20 mg/kg of L-DOPA (MPTP acute L-DOPA; n = 6), or 20 mg/kg of L-DOPA twice daily for three months (MPTP chronic L-DOPA; n = 12). Six animals developed severe and reproducible dyskinesias, presenting choreic–athetoid (characterized by constant writhing and jerking motions), dystonic and sometimes ballistic movements (large-amplitude flinging, flailing movements) (MPTP-intoxicated and chronically L-DOPA-treated, dyskinetic animal group), while 6 other did not (MPTP-intoxicated and chronically L-DOPA-treated, non-dyskinetic animal group). The MPTP intoxication protocol, the chronic L-DOPA treatment, the clinical assessments, the terminal procedure and the characterization of the extent of nigrostriatal denervation were conducted as previously published [3], [11], [12], [13], [14]. DAT binding autoradiography using [^125^I]-(E)-N-(3-iodoprop-2-enyl)-2β-carboxymethyl-3β-(4′-methylphenyl)-nortropane; Chelatec, France) showed a dramatic and similar reduction (>95%) in all MPTP-treated groups in comparison to control animals, as published elsewhere [12].

### Behavioural assessment

Animal behaviour was assessed in their home cages. All observers were blinded with regard to the experimental protocol. During each session, two blinded examiners evaluated the level of motor performance of each animal, by coaxing them to perform various tasks by offering appetizing fruits. Animals received supplemental feeding from day 7 onwards to maintain their body weight as constant as possible. The degree of parkinsonism was assessed daily (9 a.m.) for 30 min using a validated parkinsonian macaque clinical scale [3], [11], [12], [13], [14] which rates the following symptoms of parkinsonian disability: tremor, variations in the general level of activity, body posture (flexion of spine), vocalization, freezing and frequency of arm movements (reaching for food for each upper limb) and rigidity (for each upper limb). The minimal disability score was 0 and the maximum score was 25. The severity of dyskinesia was rated using the Dyskinesia Disability Scale: 0, dyskinesia absent; 1, mild, fleeting, and rare dyskinetic postures and movements; 2, moderate, more prominent abnormal movements, but not interfering significantly with normal behaviour; 3, marked, frequent and, at times, continuous dyskinesia intruding on the normal repertoire of activity; or, 4, severe, virtually continuous dyskinetic activity, disabling to the animal and replacing normal behaviour. Median rating scores and SEM were calculated daily for each group.

### Western Blotting

Patches collected from 300 µm-thick fresh frozen coronal sections containing dorsal motor putamen were sonicated in 1% SDS and Western blotting was performed as described [6], using antibodies against phospho-Thr34-DARPP-32 [15], phospho-Ser845-GluR1 (PhosphoSolutions, Aurora, CO, USA), phospho-Thr202/Tyr204-ERK1/2 and phospho-Ser235/236-S6 (Cell Signaling Technology), and against total DARPP-32 [15], S6, ERK and GluR1 (Cell Signaling Technology). Detection was based on fluorescent secondary antibody binding and quantified using a Li-Cor Odyssey infrared fluorescent detection system (Li-Cor, Lincoln, NE). The levels of each phosphoprotein were normalized for the amount of the corresponding total protein detected in the sample.

## Results

### Parkinsonism and dyskinesia status

MPTP-treated animals displayed the classic progression of symptoms previously shown with this intoxication regimen [10]. Monkeys became increasingly bradykinetic, adopted a stooped posture, with increased rigidity of the limbs and decreased vocalization. Movement accuracy progressively deteriorated and there were occasional episodes of freezing as well as postural tremor. Stable parkinsonism was obtained after 19±0.5 MPTP injections (parkinsonian score  = 9.3±0.34). All MPTP groups displayed a similar parkinsonian score (MPTP: 8.5±0.5, MPTP acute L-Dopa: 9.5±0.5, MPTP chronic L-Dopa (dyskinetic): 9.33±0.4, MPTP chronic L-Dopa (non dyskinetic): 9.83±0.87). Parkinsonian scores of all MPTP groups were significantly different from controls (F_6,35_  = 88.12, p<0.0001) and identical between them. Animals who developed L-Dopa induced dyskinesia following chronic L-Dopa treatment had a dyskinetic score of 2.33±0.21.

### cAMP signalling

**Figure 2:**
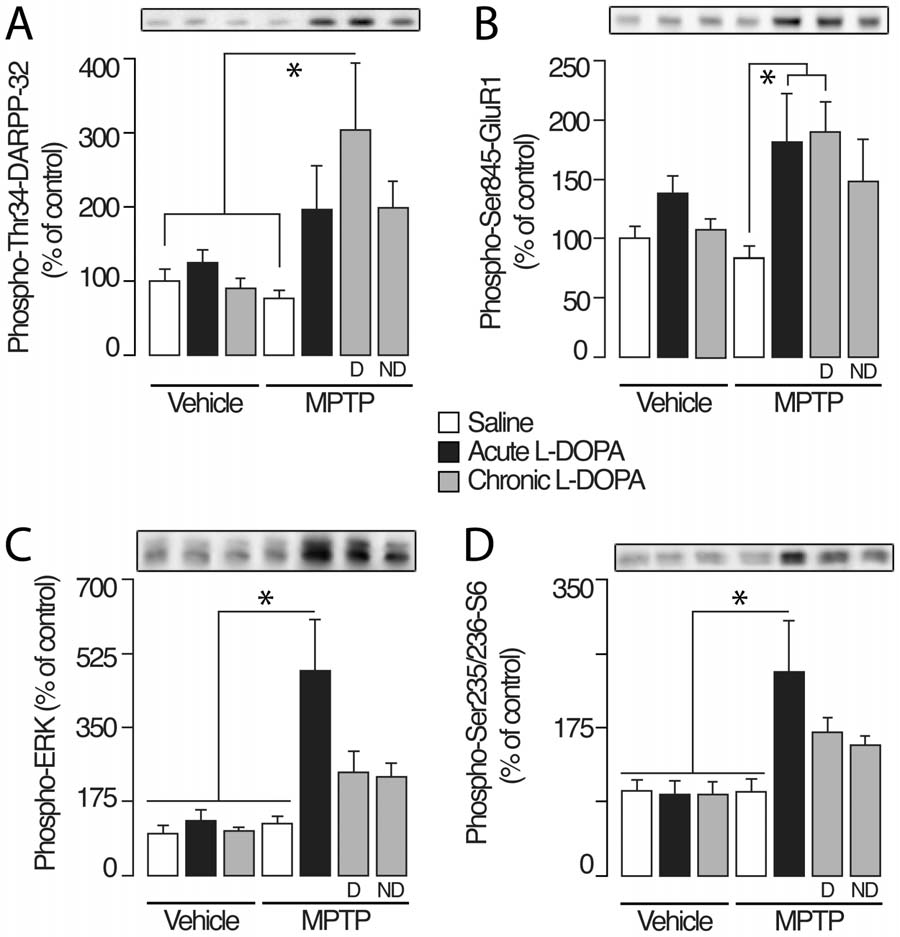
Western blot analysis of levels of (A) phosphorylated DARPP-32 at Thr34, (B) phosphorylated GluR1 at Ser845, (C) phosphorylated ERK1/2 at Thr202/Tyr204 and (D) phosphorylated S6 at Ser235/Ser236 in the dorsal putamen (mean ± S.E.M.). Groups were prepared as depicted in figure 1. Representative blots are shown above each panel. Data were analysed using a one-way ANOVA followed by post hoc Tukey-Kramer multiple comparisons test (Graphpad Instat, Graphpad softwares, San Diego, CA). A probability level of 5% (*: P<0.05) was considered statistically significant. (**A**) F(_6,37_) = 3.6, P = 0.008; (**B**) F(_6,42_) = 3.4, P = 0.008; (**C**) F(6,_38_) = 3.9, P = 0.004; (**D**) F(_6,38_) = 3.9, P = 0.004.

Administration of L-DOPA, or MPTP-induced lesion, did not alter, *per se*, the phosphorylation of DARPP-32 at Thr34 (Fig. 2A), nor did they affect the phosphorylation of another PKA target, the GluR1 subunit of the glutamate AMPA receptor (Fig. 2B). In contrast, the first ever administration of L-DOPA to MPTP-lesioned monkeys induced a trend toward increased phosphorylation of DARPP-32 at Thr34, and a significant increase in phosphorylation of GluR1 at Ser845 (Fig. 2A–B). Following chronic treatment with L-DOPA, the phosphorylation of DARPP-32 at Thr34 and of GluR1 at the PKA site, Ser845 [16], was dramatically increased in MPTP dyskinetic monkeys (Fig. 2A–B). Enhanced DARPP-32 and GluR1 phosphorylation was also detected in non-dyskinetic animals. However, these increases did not reach statistical significance (Fig. 2A–B). Total levels of DARPP-32 and GluR1 were unaffected by MPTP lesion and/or treatment with L-DOPA (Fig. 2A–B). Thus, dopamine depletion is accompanied by a considerable increase in dopamine D1 receptor responsiveness at the postsynaptic level. This supports the idea of a correlation between persistent activation of PKA signalling and severity of LID.

### ERK signalling

We next examined whether changes in phosphorylation of ERK1/2 were associated with the first ever and/or the chronic L-DOPA administration. Acute or chronic administration of L-DOPA to control monkeys, as well as MPTP lesion *per se*, did not affect ERK1/2 phosphorylation (Fig. 2C). L-DOPA produced a large (4-fold) increase in phospho-ERK1/2, when given for the first time ever to MPTP-lesioned monkeys (Fig. 2C). However, this effect was reduced to a 2.5-fold increase over control after several months of L-DOPA administration with no difference between dyskinetic and non-dyskinetic monkeys (Fig. 2C).

**Figure 3:**
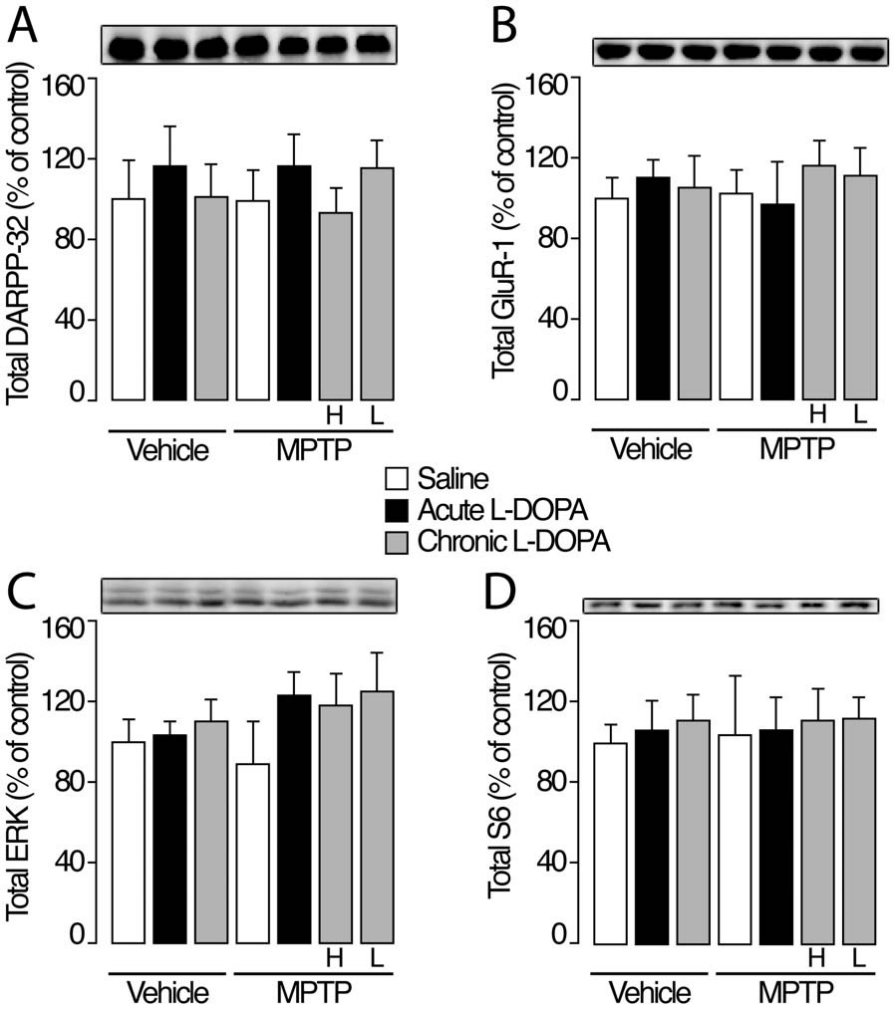
Western blot analysis of levels of (A) DARPP-32 (B) GluR1 (C) ERK1/2 and (D) S6 in the dorsal putamen (mean ± S.E.M.). Groups were prepared as depicted in Figure 1. Representative blots are shown above each panel.

Previous work showed that activated ERK phosphorylates S6 at Ser235/236 and that this regulation promotes 5′ cap-dependent initiation of messenger RNA translation [17]. In the mouse, increased S6 phosphorylation has been linked to ERK activation and to the development of LID [18]. In our experiment, the pattern of S6 phosphorylation at Ser235/236 in the various experimental groups exactly matched that of phosphorylated ERK (Fig. 2D). Total levels of ERK and S6 were unaffected by MPTP lesion and/or treatment with L-DOPA (Fig. 3C–D).

## Discussion

This study shows that, in a non-human primate model of dyskinesia, the concomitant enhancement of cAMP and ERK signalling in the dorsal striatum is associated to the induction of LIDs. Moreover, once LIDs have been established, there is a preponderant activation of the cAMP/DARPP-32 signalling cascade associated to their acute manifestation.

Acute and chronic administration regimens of L-DOPA to unlesioned monkeys do not affect the state of phosphorylation of DARPP-32 and its target, GluR1. Similarly, L-DOPA *per se* does not affect ERK and S6 phosphorylation. In contrast, after dopamine denervation, the first ever administration of L-DOPA activates both signalling cascades, as evidenced by the significant increases in the levels of phospho-Ser845–GluR1, phospho-Thr202/Tyr204-ERK and phospho-Ser235/Ser236–S6 (Fig. 2). These changes in responsiveness are most likely attributable to enhanced sensitivity of D1Rs, which has been described in rodent [6], [19] and non-human primate models [3], as well as in post-mortem samples of parkinsonian patients [20].

The increase in phosphorylation of DARPP-32 and GluR1 produced by L-DOPA following MPTP-lesion is further increased by chronic administration, but only in dyskinetic monkeys. These results suggest that sensitized cAMP signalling plays a pivotal role in the manifestation of dyskinesia. Moreover, they are in agreement with data obtained in rodents, showing that the ability of L-DOPA to increase DARPP-32 phosphorylation is preserved in dyskinetic, but lost in non-dyskinetic animals [6], [21].

The ability of L-DOPA to promote ERK signalling is maximal at the first drug administration and declines during chronic treatment. Thus, after three months of daily L-DOPA injections, the phosphorylation of ERK1/2 and S6, albeit still enhanced, is much closer to basal levels. The ability of prolonged administration to attenuate the effects of L-DOPA on ERK signalling has been previously documented in the mouse [6]. However, studies in rodent models show a correlation between LIDs and increased ERK activation [5], [6], [7]. In contrast, the present data indicate that, following prolonged L-DOPA administration, the levels of phospho-Thr202/Tyr204-ERK and phospho-Ser235/236-S6 found in dyskinetic and non-dyskinetic monkeys are undistinguishable. This discrepancy may be explained by species difference, or by the different length of exposure to chronic L-DOPA employed in the two models (10 to 21 days in rodents vs. three months in monkeys). Furthermore, as protein phosphorylation has here been determined at the single time point of 60 min after administration of L-DOPA, we cannot exclude that the effect of L-DOPA is reduced to a rapid and transient increase in ERK signalling, which cannot be detected under the present experimental conditions. Nevertheless, even this type of change would indicate an extinction of response, as a result of a prolonged administration of L-DOPA.

In conclusion, this study shows that, in non-human primates, dopamine depletion confers to L-DOPA the ability to activate the PKA/DARPP-32 and ERK cascades and that long-term (three months) administration of L-DOPA leads to regression of ERK activation, whereas cAMP/DARPP-32 signalling persists unchanged, or even exacerbated. Given the involvement of ERK and DARPP-32 in LIDs, as indicated by studies performed in rodent models [5], [6], [7], these results are compatible with the hypothesis that coordinated activation of cAMP/PKA/DARPP-32 and ERK is implicated in the priming processes underlying the emergence of dyskinesia. In this initial phase, activation of the Ras-ERK signalling cascade would lead to transcriptional/translational events necessary to produce and stabilize dyskinetic behaviour. In a later phase, once LIDs have been established, hyper-activation of PKA/DARPP-32 may become sufficient to maintain their expression independently of ERK, most likely by short-term regulation of neuronal activity through, for instance, regulation of the state of phosphorylation of GluR1 and other ion channels.

## References

[1] Cotzias GC, Papavasiliou PS, Gellene R (1969). Modification of Parkinsonism–chronic treatment with L-dopa.. N Engl J Med.

[2] Jenner P (2008). Molecular mechanisms of L-DOPA-induced dyskinesia.. Nat Rev Neurosci.

[3] Aubert I, Guigoni C, Hakansson K, Qin L, Dovero S (2005). Increased D1 dopamine receptor signalling in levodopa-induced dyskinesia.. Ann Neurol.

[4] Picconi B, Centonze D, Hakansson K, Bernardi G, Greengard P (2003). Loss of bidirectional striatal synaptic plasticity in L-DOPA-induced dyskinesia.. Nat Neurosci.

[5] Pavon N, Martin AB, Mendialdua A, Moratalla R (2006). ERK phosphorylation and FosB expression are associated with L-DOPA-induced dyskinesia in hemiparkinsonian mice.. Biol Psychiatry.

[6] Santini E, Valjent E, Usiello A, Carta M, Borgkvist A (2007). Critical involvement of cAMP/DARPP-32 and extracellular signal-regulated protein kinase signaling in L-DOPA-induced dyskinesia.. J Neurosci.

[7] Westin JE, Vercammen L, Strome EM, Konradi C, Cenci MA (2007). Spatiotemporal Pattern of Striatal ERK1/2 Phosphorylation in a Rat Model of L-DOPA-Induced Dyskinesia and the Role of Dopamine D1 Receptors.. Biol Psychiatry.

[8] Gerfen CR, Miyachi S, Paletzki R, Brown P (2002). D1 dopamine receptor supersensitivity in the dopamine-depleted striatum results from a switch in the regulation of ERK1/2/MAP kinase.. J Neurosci.

[9] Bezard E, Ferry S, Mach U, Stark H, Leriche L (2003). Attenuation of levodopa-induced dyskinesia by normalizing dopamine D3 receptor function.. Nature Med.

[10] Bezard E, Dovero S, Prunier C, Ravenscroft P, Chalon S (2001). Relationship between the appearance of symptoms and the level of nigrostriatal degeneration in a progressive MPTP-lesioned macaque model of Parkinson's disease.. J Neurosci.

[11] Nadjar A, Brotchie JM, Guigoni C, Li Q, Zhou SB (2006). Phenotype of striatofugal medium spiny neurons in parkinsonian and dyskinetic nonhuman primates: a call for a reappraisal of the functional organization of the basal ganglia.. J Neurosci.

[12] Fernagut PO, Li Q, Dovero S, Chan P, Wu T (2010). Dopamine transporter binding is unaffected by chronic L-DOPA treatment: relevance to imaging studies. submitted..

[13] Ahmed MR, Berthet A, Bychkov E, Porras G, Li Q (2010). Lentiviral overexpression of GRK6 alleviates L-dopa-induced dyskinesia in experimental Parkinson's disease.. Sci Transl Med.

[14] Berton O, Guigoni C, Li Q, Bioulac BH, Aubert I (2009). Striatal overexpression of DeltaJunD resets L-DOPA-induced dyskinesia in a primate model of Parkinson disease.. Biol Psychiatry.

[15] Snyder GL, Girault JA, Chen JY, Czernik AJ, Kebabian JW (1992). Phosphorylation of DARPP-32 and protein phosphatase inhibitor-1 in rat choroid plexus: regulation by factors other than dopamine.. J Neurosci.

[16] Roche KW, O'Brien RJ, Mammen AL, Bernhardt J, Huganir RL (1996). Characterization of multiple phosphorylation sites on the AMPA receptor GluR1 subunit.. Neuron.

[17] Roux PP, Shahbazian D, Vu H, Holz MK, Cohen MS (2007). RAS/ERK signaling promotes site-specific ribosomal protein S6 phosphorylation via RSK and stimulates cap-dependent translation.. J Biol Chem.

[18] Santini E, Heiman M, Greengard P, Valjent E, Fisone G (2009). Inhibition of mTOR signaling in Parkinson's disease prevents L-DOPA-induced dyskinesia.. Sci Signal.

[19] Santini E, Alcacer C, Cacciatore S, Heiman M, Herve D (2009). L-DOPA activates ERK signaling and phosphorylates histone H3 in the striatonigral medium spiny neurons of hemiparkinsonian mice.. J Neurochem.

[20] Corvol JC, Muriel MP, Valjent E, Feger J, Hanoun N (2004). Persistent increase in olfactory type G-protein alpha subunit levels may underlie D1 receptor functional hypersensitivity in Parkinson disease.. J Neurosci.

[21] Kim DS, Palmiter RD, Cummins A, Gerfen CR (2006). Reversal of supersensitive striatal dopamine D(1) receptor signaling and extracellular signal-regulated kinase activity in dopamine-deficient mice.. Neuroscience.

